# The relationship between executive functions and mathematics: a systematic review with meta-analysis of longitudinal studies

**DOI:** 10.1186/s41155-025-00362-1

**Published:** 2026-01-22

**Authors:** Pedro Paulo Marci Tette, Cláudia Nascimento Guaraldo Justi, Francis Ricardo dos Reis Justi

**Affiliations:** 1 Graduate Program in Psychology, Cognition and Language Research Group, Federal University of Juiz de Fora, Juiz de Fora, Brazil; 2 Department of Psychology, Cognition and Language Research Group, Federal University of Juiz de Fora, Juiz de Fora, Brazil

**Keywords:** Executive function, Math, Meta-analysis, Longitudinal

## Abstract

**Objective:**

This study examined the relationship between executive functions (EF) and mathematical skills throughout development using a meta-analysis of longitudinal studies.

**Method:**

This study included (a) longitudinal studies that (b) reported correlations between EF measures (assessed at Time 1) and mathematics outcomes (assessed at Time 2) in (c) typically developing samples ranging in age from birth to 18 years. Studies were excluded if they were (a) not written in English or Portuguese, (b) aggregated data from typical and atypical populations, or (c) combined data from children and adolescents without distinction. A systematic search was conducted in October 2021 and later updated in 2025 using PsycINFO, SciELO, and PubMed. The risk of publication bias was assessed using funnel plot analysis and Egger’s test. A random-effects meta-analysis was performed.

**Results:**

Twenty-nine studies involving children and adolescents (*n* = 104,295; M_age at Time 1 = 5.4 years; M_age at Time 2 = 8.4 years) were included. The overall correlation between EF and mathematics was moderate and statistically significant (*r* = 0.30, 95% CI [0.24, 0.36]). Among EF components, working memory showed the strongest association with mathematical performance (*r* = 0.43, 95% CI [0.35, 0.50]), followed by cognitive flexibility (*r* = 0.34, 95% CI [0.27, 0.42]) and inhibitory control (*r* = 0.21, 95% CI [0.13, 0.29]). Age and study quality did not significantly moderate the relationship between EF and mathematics.

**Conclusion:**

The findings suggest that EF, particularly working memory, is a meaningful predictor of mathematical performance across development. These results underscore the importance of early EF assessment in informing interventions designed to prevent math learning difficulties. Despite the low risk of publication bias, the high heterogeneity observed in most analyses suggests the influence of additional moderating variables that warrant further investigation.

**Supplementary Information:**

The online version contains supplementary material available at 10.1186/s41155-025-00362-1.

## Introduction

Mathematics is fundamental to understanding the world around us. Numerical language, developed throughout history, has made it easier to understand quantities through counting and calculations. Despite the importance of mathematics in our lives, the underlying processes of learning and performing mathematics still need to be better understood. It can be said that the development of mathematics is based on both domain-specific cognitive processes, such as non-symbolic and symbolic representations of numerosity (Siegler, 2022), and domain-general cognitive processes, such as executive functions (EF), for example (Noël & Karagiannakis, 2022).

Considering general cognitive processes, EF can be understood as a set of complex top-down mental processes essential for carrying out activities requiring concentration and attention (Diamond, 2013). This system runs in the prefrontal regions of the brain and acts by managing and operationalizing other processes, systems, and skills. EFs are resource-demanding processes that allow us to control and monitor thoughts and behaviors to provide desired responses to defined goals (Bellon et al., 2019; Cragg & Gilmore, 2014; Diamond, 2013).

Although studies have shown that EFs are important for performance in mathematical activities (see, for example, the meta-analysis by Spiegel et al., 2021), how this occurs still needs to be determined. After all, what is the magnitude of this relationship? Is the relationship stable throughout development? In addition, questions about the nature of EFs and different ways to assess them make those questions more complex.

As for the nature of EF, Miyake et al. (2000), using confirmatory factor analysis, observed the existence of three components of EF that would be distinguishable but not independent, as they share common underlying processes: mental set shifting (“Shifting”), information updating and monitoring (“Updating”), and inhibition of preponderant responses (“Inhibition”). Expanding on these findings, Diamond (2013) proposed a widely adopted and comprehensive framework that reorganizes these components into: 1) *inhibitory control* (IC), described as the deliberate ability to inhibit dominant responses in order to suppress distractions and unwanted reactions to achieve a goal; 2) *cognitive flexibility* (CF), defined as the ability to switch flexibly between mental sets, tasks and goals; and 3) *working memory* (WM), understood as an ability to retain, monitor and manipulate mental information for a short interval of time in order to carry out some activity (Diamond, 2013; Miyake & Friedman, 2012; Miyake et al., 2000).

Although most of the studies carried out on EF are based on the model proposed by Miyake et al. (2000), it cannot be said that there is a consensus in the literature. Competing factorial models—including unidimensional, shifting-updating merged, inhibition-updating merged, and bifactor frameworks (Karr et al., 2018)—continue to be investigated, often combining or omitting core components such as IC, CF, and WM.

In this sense, Karr et al., (2018) observed that developmental stage appears to play a critical role in model fit, for example: while one- or two-factor models better represent preschool populations (Blums et al., 2017; Van der Ven et al., 2012; Simanowski & Krajewski, 2019), three-factor or nested models are more appropriate for school-aged children (Fuhs et al., 2016; Michel et al., 2020; Morgan et al., 2019) and adolescents (Ahmed et al., 2019; Watts et al., 2015). This variability likely reflects the gradual differentiation of EF components across the lifespan, with CF being the last to fully mature (Davidson et al., 2006; Diamond, 2013). As a result, conclusions regarding the"optimal"EF model should be interpreted with caution, given that age and task demands significantly influence structural validity (Doebel, 2020; Karr et al., 2018). Moreover, methodological choices—such as selecting unidimensional versus multicomponent frameworks—may further contribute to inconsistencies across studies, as WM, IC, and CF play distinct roles depending on how they are operationalized.

In addition to the conceptualization of executive functions (EF), another critical factor to consider is the type of task used to assess them. The literature demonstrates considerable variability in the instruments employed, with evidence suggesting that tasks such as the Tower of London, Tower of Hanoi, and Random Number Generation task concurrently engage multiple EF components. These are, therefore, classified as complex EF tasks, as they recruit a range of executive processes simultaneously (Bull & Lee, 2014; Miyake et al., 2000; Welsh et al., 1999), making it difficult to isolate the unique contribution of individual components in studies that rely on these measures. Results from correlational and regression analyses in studies using the tower tasks (Bull et al., 2004; Welsh et al., 1999), as well as findings from structural equation modeling (SEM) in studies involving the Random Number Generation task (Miyake et al., 2000), indicate that these instruments activate distinct and overlapping executive processes. As a consequence, there is no clear consensus in the literature regarding which specific EF components are primarily involved in each task (Batista et al., 2007; Bull & Lee, 2014; Bull et al., 2004; Welsh et al., 1999). As warned by Bull et al. (2004), although it is unlikely that an instrument will be able to measure a single component of EF due to the variance shared among them, the magnitude of the relationship between the indexed components must not indicate redundancy of the measures. On the other hand, some instruments capture predominantly one component of EF, such as IC in the Stroop task (Archibald & Kerns, 1999; Gerstadt et al., 1994; Roebers et al., 2011), WM in the Backwards Digit Span (Alloway, 2007; Alloway et al., 2006; Blackwell, 2001; Woodcock et al., 2001) and CF in the Dimensional Change Card Sorting task (Zelazo, 2003; 2006).

Different reviews with meta-analysis have investigated the relationship between EF and performance in academic activities (Allan et al., 2014; Chen & Bailey, 2021; Friso-van den Bos et al., 2013; Pascual et al., 2019; Peng et al., 2016; Santana et al., 2022; Spiegel et al., 2021; Yeniad et al., 2013; Zhong et al., 2022). EF has generally been considered a good predictor of academic performance in children and adolescents. Specifically, it has been noted that EF plays a role in mathematical performance. For example, when solving mathematical problems, information needs to be stored, retrieved, and interleaved in WM (e.g., keeping the number"5"while retrieving the number"6"in the sum"5 + 6"); flexible strategies for switching between information need to be applied (e.g., alternating between Arabic symbols, verbal numerical codes and non-symbolic representation) and the inhibition of erroneous responses and other dominant strategies (e.g. addition when subtraction is required) is also necessary (Bull & Lee, 2014; Van der Ven et al., 2012).

Considering WM, WM is the EF component most strongly associated with mathematics (Friso-van den Bos et al., 2013; Peng et al., 2016; Spiegel et al., 2021). Meta-analyses have found a moderate and significant correlation between WM and mathematics (*r* = 0.38, Friso-van den Bos et al., 2013; *r* = 0.39, 95% CI [0.36, 0.41], Spiegel et al., 2021; *r* = 0.35, 95% CI [0.32, 0.37], Peng et al., 2016). Studies suggest that sample characteristics, age, and the type of math test moderate this relationship. Regarding sample characteristics, Friso-van den Bos et al. (2013) and Peng et al. (2016) found that the correlation between WM and mathematics was stronger in individuals with cognitive deficits compared to those with typical development. Concerning age, Peng et al. (2016) reported stronger associations between WM and calculation as well as WM and geometry in younger children. Similarly, Friso-van den Bos et al. (2013) found that age (4–12 years) moderated the relationship between visuospatial WM and mathematics, with older children showing weaker correlations. Nevertheless, age did not affect the relationship between verbal WM and mathematics. The WM-mathematics relationship appears to be stable from early childhood through adolescence. However, longitudinal studies found no moderating effect of age (5–15 years) on problem-solving and division/fraction tasks after controlling for prior academic achievement and socioeconomic status (Ahmed et al., 2019; Watts et al., 2015). In contrast, Stipek and Valentino (2015) tested the fade-out hypothesis, observing that while WM predicts mathematical performance in early school years (ages 5–9), this association weakens by adolescence (ages 9–14) in general mathematics assessments. Similarly, Simanowski and Krajewski (2019) found no age-related moderation in the WM-mathematics relationship for diverse skills (e.g., arithmetic fluency, general mathematics tests) in children aged 5–8. The relationship between WM and mathematics may also depend on the type of mathematical test used. Peng et al. (2016) found that WM correlated more strongly with word-problem solving (*r* = 0.37, 95% CI [0.34, 0.41]) and calculation (*r* = 0.35, 95% CI [0.32, 0.39]) but more weakly with geometry (*r* = 0.23, 95% CI [0.17, 0.27]). In the same way, Spiegel et al. (2021) reported the strongest correlation between WM and word-problem solving (*r* = 0.43, 95% CI [0.40, 0.47]), followed by calculation (*r* = 0.37, 95% CI [0.34, 0.40]) and mathematical fluency (*r* = 0.29, 95% CI [0.25, 0.33]). Furthermore, Friso-van den Bos et al. (2013) found that general mathematical measures correlated more strongly with WM than specific tests (e.g., arithmetic, counting, numerical knowledge, word-problem solving, and geometry). Despite these findings, discrepancies remain. While Peng et al. (2016) found no significant difference between the WM correlations for word-problem solving and calculation, Spiegel et al. (2021) reported a stronger association with word-problem solving.

In summary, working memory demonstrates a robust and consistent association with mathematical performance across developmental stages, with meta-analytic correlations ranging from *r* = 0.35 to 0.39. This relationship is moderated by sample characteristics (e.g., effects in children with cognitive deficits) and the type of mathematical task (e.g., correlations with problem-solving and calculation rather than fluency or geometry). However, the role of age remains contested: while some studies suggest a decline in WM’s predictive power with age (e.g., Stipek & Valentino, 2015), others report stability (e.g., Ahmed et al., 2019) or no moderation (e.g., Simanowski & Krajewski, 2019). Further research is required to resolve these inconsistencies, particularly in understanding how different mathematical tasks influence the WM-mathematics relationship.

Concerning CF, the meta-analysis literature has reported moderate and significant effect sizes between CF and mathematical performance in children (*r* = 0.28, Friso-van den Bos et al., 2013; *r* = 0.35, 95% CI [0.34, 0.36], Santana et al., 2022; *r* = 0.26, 95% CI [0.15, 0.35], Yeniad et al., 2013). However, the role of potential moderating variables in this relationship remains unclear. For instance, Yeniad et al. (2013) found no significant moderators, such as CF test type, test design, age, educational level, socioeconomic status, intelligence, or gender. In contrast, Santana et al. (2022) stratified their sample into three age groups (4.75–5, 5–8, and 9–12 years) and observed that the effect of CF on mathematical performance decreased with age. Although age was identified as a moderating factor, the specific mathematical domains assessed were not. Individual studies highlight CF’s role in mathematics. For example, Morgan et al. (2019) demonstrated that CF predicts performance even after controlling for prior achievement and socioeconomic status in early school years. Nevertheless, this relationship is context-dependent. Fuhs et al. (2016) found that early numerical skills mediated the CF-mathematics link in kindergarten. However, CF directly predicted problem-solving tasks by second grade, suggesting task complexity amplifies its role. In contrast, Simanowski and Krajewski (2019) reported that when early numerical competencies were controlled, CF was no longer a significant predictor of basic numerical skills, arithmetic, or general mathematics performance. Similarly, Van der Ven et al. (2012) found no predictive effect of CF when WM was included as a moderator. Methodological choices also shape interpretations. In particular, some studies have combined CF and IC into a single factor, which may influence how their effects on mathematical performance are interpreted (Fuhs et al., 2016; Simanowski & Krajewski, 2019; Van der Ven et al., 2012). Additionally, while age did not consistently moderate the CF-mathematics association (Fuhs et al., 2016; Morgan et al., 2019), Morgan et al. (2019) noted age’s direct effect on mathematical performance. In the meta-analysis conducted by Spiegel et al. (2021), CF correlated more strongly with word-problem solving (*r* = 0.35, 95% CI [0.22, 0.47]) and calculation (*r* = 0.29, 95% CI [0.19, 0.39]) than with math fluency (*r* = 0.16, 95% CI [0.08, 0.23]), indicating that the type of mathematical skill assessed influences the strength of the CF-math relationship.

In summary, CF demonstrates moderate but significant associations with mathematical performance (*r* = 0.26–0.35) (Friso-van den Bos et al., 2013; Santana et al., 2022; Yeniad et al., 2013), particularly in problem-solving and calculation tasks. Nevertheless, the role of moderators like age and mathematical domains remains contested. While some studies suggest CF’s influence diminishes with age (Santana et al., 2022), others find no consistent age-related moderation (Fuhs et al., 2016; Morgan et al., 2019). Furthermore, CF correlates more strongly with complex mathematical skills (e.g., word problems) than with basic fluency (Spiegel et al., 2021), highlighting the role of task complexity. Thus, although studies highlight the importance of CF for mathematical performance, whether age and different mathematical domains moderate this relationship remains uncertain.

Regarding IC, Zhong et al. (2022), in a meta-analysis of studies with a Chinese sample, found a significant correlation between IC and mathematics in preschool children (*r* = 0.35, 95% CI [0.22, 0.46]). The authors identified moderating effects of IC measurement type, gender, and age. Specifically, the IC-math correlation was stronger in younger children (4–5 years: *r* = 0.42, 95% CI [0.34, 0.50]) than in older preschoolers (5–6 years: *r* = 0.28, 95% CI [0.17, 0.37]). In contrast, Allan et al. (2014) reported similar correlation levels in preschoolers (*r* = 0.29, 95% CI [0.24, 0.32]) and kindergarteners (*r* = 0.27, 95% CI [0.22, 0.33]), suggesting a consistent relationship between IC and mathematics throughout early childhood. Similarly, Spiegel et al. (2021) and Zhu et al. (2025) found no significant moderating effect of age on the IC-math relationship from kindergarten to sixth grade. The literature also presents inconsistent findings regarding the moderating role of math task type. For instance, Viterbori et al. (2015) found no significant correlation between IC and calculation but observed a stronger link between IC and word-problem solving. In contrast, Blums et al. (2017) reported a significant correlation between IC and both types of tasks, suggesting that the specific mathematical domain assessed may shape the IC-math relationship.

In summary, IC exhibits a dynamic relationship with mathematical development: while some evidence suggests that age moderates this relationship (Michel et al., 2020), broader analyses indicate no consistent age-related effects (Spiegel et al., 2021; Simanowski & Krajewski, 2019). The type of mathematical task further complicates the relationship: some studies report a stronger IC correlation with problem-solving than with calculation (Viterbori et al., 2015), while others find consistent correlations across both task types (Blums et al., 2017). The lack of consensus on moderators such as age and task type underscores the complexity of the IC-math relationship.

An analysis of the literature shows that EFs are fundamental for math performance. Different meta-analyses have been carried out on the relationship between WM and mathematics (Friso-van den Bos et al., 2013; Peng et al., 2016), between IC and mathematics (Allan et al., 2014; Zhu et al., 2025), between CF and mathematics (Santana et al., 2022; Yeniad et al., 2013), as well as between the three components of EF and math performance (Pascual et al., 2019; Spiegel et al., 2021; Zhong et al., 2022), but, as we have seen, these are inconsistent with each other. Although the three components of EF correlate significantly with mathematics, it still needs to be determined whether age and different types of mathematical skills are moderators of this relationship. Another crucial issue is the direction of the relationship between EF and math; despite suggesting that EF contributes to math, all of these meta-analyses are based on cross-sectional or mixed cross-sectional with longitudinal data. However, longitudinal data showing that past EF predicts future math performance is necessary to affirm that EF predicts math performance.

Given the inconsistencies in the past studies, this study investigated the relationship between the three components of EF and mathematics using a meta-analysis that considered age and the type of math test as potential moderators of this relationship. An essential aspect of this meta-analysis is that it included only longitudinal studies and considered only the correlations between EF measured at time 1 (past) and math measures measured at time 2 (future), thus allowing us to infer whether EFs can predict future math performance. Considering the importance of the developmental perspective when seeking to understand the relationship between EF and mathematics, it is noteworthy that we did not find studies like this in the literature.

## Method

### Inclusion and exclusion criteria

The following inclusion criteria were used: a) studies with a longitudinal design; b) studies that empirically evaluated the relationship between EF and math performance; c) studies with typically developing samples; d) studies with an age range of birth to 18 years; e) studies that reported statistical data indicating a correlation between some measure of EF and math performance.

The following exclusion criteria were used: a) studies written in languages other than Portuguese and English; b) studies that presented data from samples with typical and atypical development in aggregate form; c) studies that analyzed data from children and adolescents in aggregate form; d) materials without open access on the internet or via Capes Periodicals Portal, book chapters, dissertations, theses and abstracts of papers presented at congresses.

### Search strategies

**Fig. 1:**
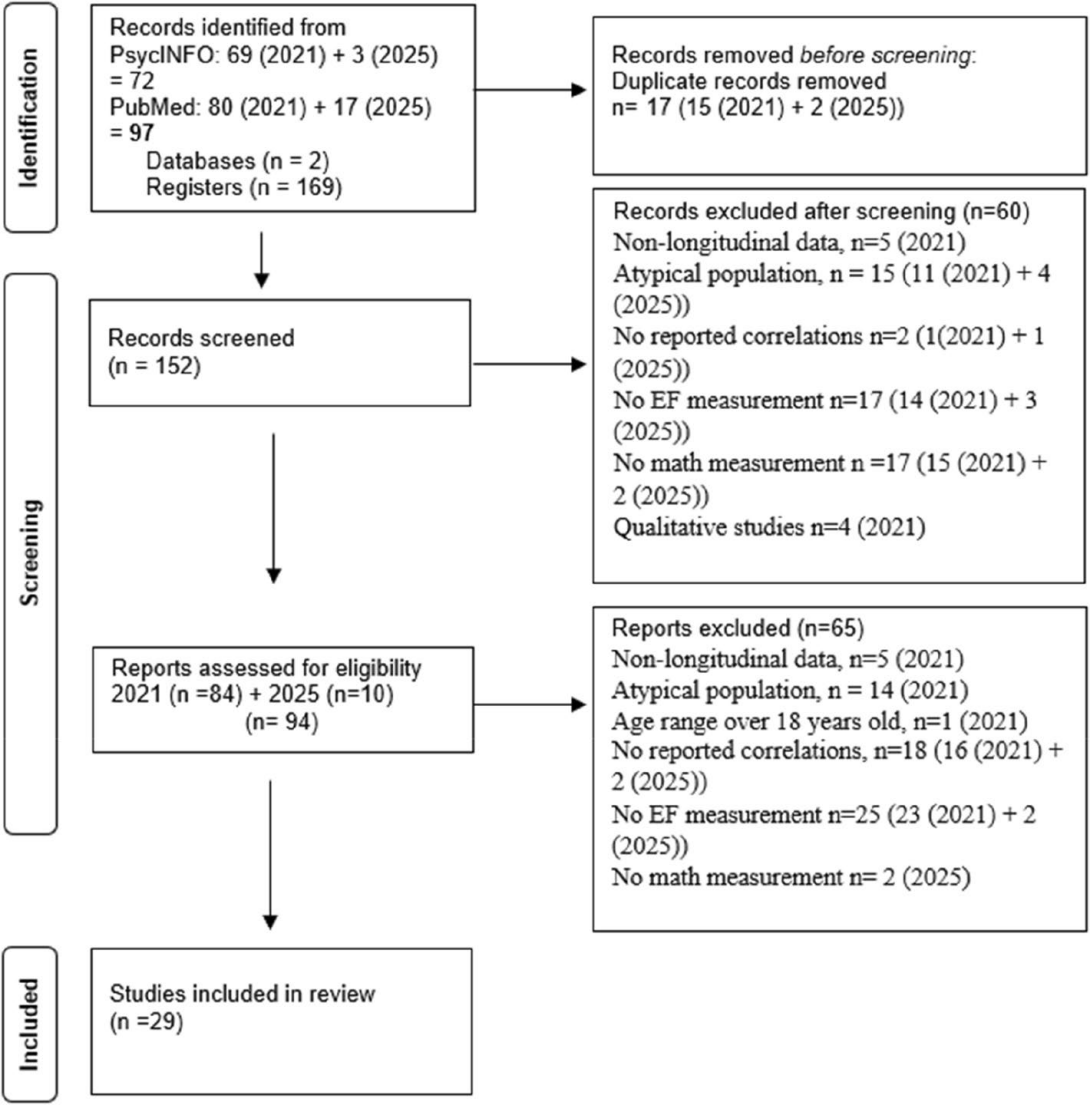
Flowchart of the Selection of Articles and Studies Reviewed

An electronic search was conducted in October 2021 across three databases—PsycINFO, Scielo, and PubMed—via the Capes Periodicals Portal. The search terms"math","executive function", and"longitudinal"were used with the Boolean operator"AND". In addition, the study was conducted according to the PRISMA guidelines (Preferred Reporting Items for Systematic Reviews and Meta-Analyses—Galvão et al., 2022) for systematic reviews. In 2025, the same search was repeated to update the meta-analysis with recent literature. As illustrated in Fig. 1, the 2021 search identified 169 articles (69 from PsycINFO, 80 from PubMed), while the 2025 search yielded an additional 20 articles (3 from PsycINFO, 17 from PubMed). After removing 17 duplicates (15 from 2021 and 2 from 2025), the remaining 152 records were screened. Titles, abstracts, and a rapid full-text scan were used to apply inclusion/exclusion criteria. At this stage, 60 articles were not included: 5 for not fitting inclusion criterion'a'(studies with a longitudinal design in 2021); 4 for criterion'b'(studies that empirically evaluated the relationship between EF and math performance in 2021); 15 for criterion'c'(studies with typically developing samples: 11 in 2021, 4 in 2025); and 36 for criterion'e'(studies that reported statistical data indicating a correlation between some measure of EF and math performance: 30 in 2021, 6 in 2025). This left 94 articles (84 from 2021, 10 from 2025) for full-text eligibility assessment.

During the full-text eligibility assessment, 60 articles were excluded: 5 articles for not meeting inclusion criterion'a'(studies with a longitudinal design in 2021); 14 for criterion'c'(studies with samples with typical development in 2021); 1 for criterion'd'(studies with an age range of birth to 18 years in 2021); and 45 for criterion'e'(studies that reported statistical data indicating a correlation between some measure of EF and math performance: 39 in 2021, 6 in 2025). Therefore, 29 articles were included in the meta-analysis. The inclusion/exclusion process was conducted by two independent evaluators and judged by a third in case of disagreement. The third evaluator was consulted three times during the 2021 search phase.

### Quality criteria for included studies

Meta-analysis is a technique with multiple applications that combines statistical parameters obtained from different studies in order to generate a synthesis of the data (Borenstein et al., 2021). Assessing the quality of the studies included is essential to avoid bias in the analyses carried out.

The quality of each study included in the meta-analysis was assessed using eight criteria: a) Sample size > 115^1^); b) Reporting of at least two demographic variables (e.g., age, gender, socioeconomic status, maternal education); c) Clear reporting of data collection times; d) Reporting of the distribution of variables; e) Report of the reliability of the target measures; f) Measures with reliability above 0,70; g) Report of the type of correlational analysis; h) Report of the mean and standard deviation. One point was awarded for each criterion met by the study. Criteria"e"and"f"were calculated from the average of the points computed for each measure (IC, WM, CF, and math). The closer to eight points a study obtained in total, the better its quality; the closer to zero points, the worse its quality (please refer to the online supplemental material, see Appendix A).

### Coding procedures

All the studies were coded individually. Firstly, data concerning author, year of publication, objectives, design, and sample type (average age, school year, nationality) were tabulated to understand the general methodology of the studies.

Next, Diamond's (2013) definition was used to tabulate the EF measures (IC, CF, and WM), excluding data from the Hanoi and London Towers and the Random Number Generator task, due to their substantial overlap in engaging multiple EF components simultaneously (Bull et al., 2004; Miyake et al., 2000; Welsh et al., 1999). Aggregated data from the three EFs were also excluded, thus forming three categories of data: IC, CF, and WM. Based on the selected articles, the measures of mathematical performance were divided into three categories: calculation tests (where accuracy and fluency in calculation are grouped together), word-problem solving tests, and general mathematics tests (which assess various mathematical skills within a single test).

### Dealing with the problem of correlated effect-sizes

As pointed out by Hedges et al. (2010), meta-analytic techniques rely on the assumption that effect size estimates from different studies are independent. However, when more than one effect size from the same study is included in the same meta-analysis, this assumption is violated because these effect sizes are correlated. One possible solution for this problem, which was adopted in the present study, is to compute the mean effect size for each study with more than one effect size (Hedges et al., 2010). In the present meta-analysis, we adopted the following procedures to include only one effect size per study. First, the correlations were separated according to the components of EF (CF, IC, and WM) and the type of math test (calculation, word-problem solving tests, and general mathematics test), resulting in three data sets for each EF component (e.g., CF-Calculation, CF-word-problem, CF-general math test, and so on). Additionally, in each study, when more than one correlation was provided between EF components (IC, CF, and WM) at Time 1 and mathematics at later times, the means of the correlations were calculated after being transformed into Fisher's z-statistic values. After data analysis, the Fisher's z values were converted back to correlation values.

Second, a fourth data set was formed for the General Analysis: All Mathematics Tests, containing all correlations according to each EF component with all math tests combined (e.g., CF-Calculation, word-problem and general math test). Again, in each study, when more than one correlation was provided between each component of EF (IC, CF, and WM) at Time 1 and All Mathematics Tests at later times, the means of the correlations were calculated.

Third, a fifth data set was created for the General Analysis: EF and Performance in Mathematics. In this data set, the means of the correlations for the three components (labeled as EF, that is, all three EF components as a single correlation) with all three types of tests combined (labeled as Performance in Mathematics, all three mathematics tests as a single correlation) were calculated. Additionally, the average sample size, as well as the mean initial and final ages, were calculated after the means of the correlations were determined for the all-data sets, in preparation for the subsequent meta-analysis.

### Statistics analysis

The analyses were conducted using Jamovi software (version 2.3) via the Major—Meta-analysis for the Jamovi module (version 1.2). The correlations obtained from the relationship between EF and mathematical performance were transformed into Fisher-z values (Borenstein et al., 2021), and the magnitude of the effect size, the standard error, and the confidence intervals (IC) were calculated.

Considering the variations among the studies in terms of the sample's age, school level, data collection times, and instruments used to assess EF and math, the random effects model was used to conduct the analyses. This model has a more conservative approach, as it takes into account real variability in variance and effect size (Borenstein et al., 2021).

Different analyses were conducted in the study. Firstly, a general analysis included all the components of EF (IC, CF, and WM) and mathematics in general (all categories of math tests). Subsequently, analyses were carried out between each one of the EF components (IC, CF, and WM) and math measures in general. Subgroup analyses were then conducted to assess the relationship between each EF component (IC, CF, and WM) and each kind of math test (calculation, word-problem solving, and general math tests). In addition, meta-regression analyses were carried out to check whether age moderated the relationship between mathematical skills and EF and whether the quality criteria of the studies (please refer to the online supplemental material, see Appendix A) influenced the relationship.

Cohen's (2013) classification was used to assess the effect sizes. In this sense, an effect size r is considered small between 0.10 and 0.30, moderate between 0.30 and 0.50, and large when equal to or greater than 0.50.

### Heterogeneity

Two procedures were used to assess heterogeneity. The first was the Cochran test *Q* (Cochran, 1954), which takes into account the sum of the squared deviations of the effect size estimates of the studies and the effect estimate by its inverse variance. The second was the *I*^*2*^ statistic (Higgins & Thompson, 2002), which represents the percentage of the overall variance attributed to between-studies heterogeneity, with values ranging from 0 to 100%. Heterogeneity is considered low up to 25%, moderate up to 50%, and high if it exceeds 75% (Higgins et al., 2003). According to Higgins et al. (2019), heterogeneity can occur due to sampling error, real variability in variance and effect size, or a third variable as a moderator.

### Publication bias

Publication bias is the tendency to publish more studies with positive results than negative ones, and, as a consequence, this biased evidence is reflected in the final results of the meta-analysis. This bias was assessed by analyzing the funnel plot with Fisher-z values and standard deviation, as well as Egger's test, according to the method suggested by Egger et al. (1997). Bias is analyzed in the funnel plot based on the asymmetry of the data, which, when symmetrical, suggests a low probability of publication bias. The Egger test uses the regression method to test a linear association between effect size and standard error. Thus, if the p-value of Egger's test is less than 0.05, there is an indication of publication bias (Egger et al., 1997).

## Results

### General description of the studies

The database search found articles over a 17-year period (2007–2024) with increased publications from 2014 onwards (69% of the studies were published between 2014–2021). The countries of the participants of the studies were the United States (62%), Germany (7%), Australia (3%), China (3%), Italy (3%), New Zealand (3%), Norway (3%), Switzerland (3%), and 7% of the studies did not identify the nationality of the sample. The articles selected for the study included data from a representative sample of children and adolescents. The number of initial participants was 104,295 in total. The average starting age was 5.42 years [95% CI: 4.87, 5.96 years], and the average final age was 8.43 years [95% CI: 7.40, 9.45 years], as shown in Table 1.

**Table 1:** Characteristics of the studies included in the meta-analysis

Author	N initial	Mean Initial Age (SD)	Mean Final Age(SD)	School grades	Time interval		Effect Size	
						CF	IC	WM
Ahmed et al (2019)	1364	4.64	15	K*, G9*	10	x	−0.01	0.35
Blums et al. (2017)	1084	4.5	8.0*	PRE-K, G3	4*	x	0.01	x
Braak et al (2022)	287	5.8 (0.29)	10.8	K, G5	6	x	0.38	x
Bull et al. (2008)	111	4.99 (0.33)	7.71	Year 1 (K), year 2 (G1), Year 3 (G2)	2	0.15	0.33	0.30
Clark et al (2010)	108	4.0*	6.0*	PRE-K*, K*	2*	0.33	0.41	x
Clark et al. (2013)	228	2.99 (0.06)	5.25	PRE-K, K	2	x	0.22	0.23
Finders et al., 2020)^a^	18,170	5.53*	9.0*	K, G3	3	0.34	x	0.69
Finders et al. (2022)	316	4.56 (0.29)	5.06*	K	0.5	x	0.52	x
Fuhs et al (2016)	141	6.16 (0.36)	8.0*	K, G2	2	0.65	0.37	x
Hu et al. (2024)	236	10.7 (0.49)	11.7	X	1	0.24	0.05	0.26
Loningan et al., 2017)	1387	4.5 (0.42)	5.42*	PRE-K	1*	x	0,.22	x
Malone et al (2020)	569	5.32 (0.36)	6.31 (0.36)	K, G1	1	x	0.11	0.32
Mauer et al. (2023)	258	7.4	9.3	G1	1.9	x	−0.15	x
McClelland et al (2007)	310	4.48 (0.33)	4.93 (0.36)	PRE-K	0.5	x	0.39	x
Michel et al. (2020)	208	5.6 (0.56)	7.6 (0.60)	K, G1/G2	2	0.03	−0.18	0.34
Morgan et al. (2019)^a^	8920	6.0*	8.13 (0.37)	K, G1, G2	2.5	0.56	x	0.34
Nguyen & Duncan (2019)^a^	17,300	5.62 (0.37)	8.0*	K, G3	3	0.34	x	0.51
Nguyen et al (2019)^a^	18,170	5.62	7.0*	K, G1	1.5	0.35	x	0.53
Perry et al (2018)	1292	5.02(0.02)	7.95 (0.03)	K, G1, G2	3	0.34	0.3	0.32
Roebers et al (2014)	169	5.78 (0.36)	7.75	PRE-K, K, G1	2	0.45	0.31	0.51
Simanowski and Krajewski (2019)	262	5.67 (0.29)	8.25 (0.3)	K, G1, G2	3	0.23	0.19	0.24
Stipek and Valentino (2015)^c^	5873	3.0*	14.0*	PRE-K*, K*, G1* G2*,G3*, G4*, G5* G6*, G7*, G8*, G9*	11	x	x	0.3
Sung & Wicrama (2018)^a^	18,170	4.0*	8.0*	K, G1, G2	2	0.34	x	0.58
Van der Ven et al. (2012)	227	6.42(0.35)	7.42*	G1, G2	1	0.23	0.22	0.23
Virtebori et al., (2015)	206	5.70 (0.28)	8.67	Preschool (K) G1, G3	3	0.22	0.11	0.22
Vukovic et al (2014)	163	6.0 (0.33)	10.0 (0.33)	G1, G2, G4	1	x	x	0.44
Waters et al (2021)^b^	1364	4.64 (0.09)	6.0*	K*, G1	1	x	0.26	0.43
Watts et al (2015)^b^	1362	7.03 (0.02)	15.0*	G1, G2, G3, G4 G5, G6, G9*	8*	x	0.24	0.39
Willoughby et al. (2019)^a^	6040	5.61	8.11	K, G1, G2	2.5	0.31	x	0.55

*N initial* the initial sample size; Mean Initial Age = refers to the average participant age at Time 1, *SD *Standard deviations, SD are missing where not reported by the original studies, *Mean Final Age* refers to the average participant age at Time 2, *Time interval* study duration in years, *Effect Size* the mean of the reported correlations, *PRE-K* pre-kindergarten, *K *kindergarten, *G *school grade, *x* absent, * value not reported, but inferred value

^a^ECLS-K studies

^b^NICSESID studies

^c^NLSY studies

^d^Studies included in the search update of 2025

Of the 29 articles selected (see Table 1), 32% did not assess IC, 28% of the studies used two or three instruments to assess IC, and the majority (40%) used only one instrument. In total, 21 different instruments were used to assess IC, with the Stroop task (Archibald & Kerns, 1999; Gerstadt et al., 1994; Kochanska et al., 2000; Roebers et al., 2011; Van der Ven et al., 2012) and its variations being the most frequent. Concerning CF, 45% of the studies did not assess CF, 17% used two or three instruments, and the majority (38%) used only one instrument. Thirteen instruments were used to assess CF, with the Dimensional Change Card Classification task (Zelazo, 2003; 2006) being the most commonly used. Only 28% of the studies did not assess WM, 34% assessed it with two or three instruments, and 38% assessed it with just one instrument. Eighteen instruments were used to assess WM, with the Backwards Digit Span task (Alloway, 2007; Alloway et al., 2006; Blackwell, 2001; Woodcock et al., 2001) being the most frequently used. For further information, please refer to the supplementary material, see Appendix B.

Regarding math performance assessment, the instruments were divided into three categories (see Appendix B): calculations, word-problem solving, and general math tests. For calculation, seven different instruments were used: Addition and Subtraction Subtest (Grube et al., 2010); Math Fluency (Simanowski & Krajewski, 2019); Calculation Skills Battery (Subtests: written calculations and arithmetic facts, Cornoldi et al., 2002); School Performance in Mathematics (Arithmetic; Krajewski et al., 2004); Arithmetic Subtest (Wide Range Achievement Test-3—WRAT-3—Wilkinson & Robertson, 1993); Calculation Subtest of the Woodcock-Johnson Battery (WJ-R—Woodcock & Johnson, 1989); Math Fluency Subtest of the Woodcock-Johnson Battery (WJ-III, Woodcock et al., 2001); Addition Problems (Malone et al., 2020); and, Test of Basic Arithmetic and Number Skills (TOBANS; Brigstocke et al., 2016).

Three instruments were found to measure word-problem solving (see Appendix B): Applied Problems Subtest (Woodcock & Mather, 2000; Woodcock & Munoz-Sandoval, 1996; Woodcock & Johnson, 1989; Woodcock et al., 2001); Woodcock et al., 2002; Woodcock et al., 2007); Arithmetic Problem-Solving (Lucangeli et al., 1998; Virterbori et al., 2015); and, School Performance in Mathematics (Applied Arithmetic—Krajewski et al., 2004).

For the general mathematics measure, eleven different instruments were found (see Appendix B): Ani Banani Math Test (ABMT; ten Braak & Størksen, 2021); Early Childhood Longitudinal Study Kindergarten Class (ECLS-K: 2011, Tourangeau et al., 2015); Indicators of Primary School Performance (Bull et al., 2008); Test of Early Mathematics Ability (TEMA—Ginsburg & Baroody, 2003); Cito Mathematics Test (Janssen et al., 2005); Child Math Assessment (CMA—Klein & Starkey, 2004); Math Subtest (Preschool) of the Florida Voluntary Pre-Kindergarten Assessment (VPK Math—Florida Department of Education, 2012); Fractions and Division Knowledge Test of the Calculation Subtest of the Woodcock-Johnson Battery (WJ-R; Woodcock et al., 2001); School-administered tests (Hu et al., 2024); subtests of the Heidelberger Rechentest (HRT 1–4—Haffner et al., 2005); and the Peabody Individual Achievement Test (PIAT—Dunn & Marwardt, 1970).

## Meta-analysis

### General analysis: EF and performance in mathematics

Twenty-nine studies made up the final sample, and from them, the correlations were obtained between the EF, previously measured (Time 1), and math performance, subsequently measured (Time 2, 3, 4, and so on). A primary analysis was conducted containing 29 correlations (when a study presented more than one correlation, the mean correlation was calculated to ensure that only one correlation per study was included in the analysis) covering the three components of EF (IC, CF, and WM) and math (all three kinds of tests together). This analysis resulted in high heterogeneity (Q (28) = 753.0; Tau^2^ = 0.02; I^2^ = 98.46%; *p* < 0.001), and the effect size obtained was moderate and significant (r = 0.30, CI 95% [0.24, 0.36]), as can be seen in Table 2. An important result is that the meta-regression analysis that included the variable'quality of studies'as a moderator did not show evidence that'quality of studies'moderates the relationship between EF and math (B = −0.01; CI 95% [−0.07, 0.05], *p* = 0.70). Thus, it cannot be said that variations in the quality of the studies affected the results of the present meta-analysis.

**Table 2:** Effect sizes between EF components and mathematics performance

Component	Effect Size	*p*	Correlation 95% IC	Q (df)	I^2^	Tau^2^	n
General Analysis: EF and Math^a^	0.30	< 0.001	[0.24, 0.36]	753.0 (28)	98.46	0.02	29
IC and Math^a^	0.21	< 0.001	[0.13, 0.29]	231.2 (20)	93.65	0.03	21
IC and calculation	0.09	< 0.22	[−0.06, 0.24]	42.5(6)	90.8	0.03	7
IC and word-problem solving	0.24	< 0.001	[0.13, 0.34]	227.04 (11)	95.46	0.03	12
IC and general mathematics test	0.24	< 0.001	[0.17, 0.30]	15.58 (7)	64.54	0.004	8
CF and Math^a^	0.34	< 0.001	[0.27, 0.42]	674.9 (16)	98.94	0.02	16
CF and calculation	0.19	0.003	[0.06, 0.31]	6.45(3)	53.66	0.01	4
CF and word-problem solving	0.40	0.001	[0.16, 0.63]	22.4 (3)	92.11	0.05	4
CF and general mathematics test	0.36	< 0.001	[0.28, 0.44]	633.4 (9)	98.99	0.01	10
WM and Math^a^	0.43	< 0.001	[0.35, 0.50]	2140.6 (20)	98.99	0.03	21
WM and calculation	0.35	< 0.001	[0.29, 0.41]	3.6 (4)	0.05	0	5
WM and word-problem solving	0.33	< 0.001	[0.23, 0.43]	50.59 (6)	90.87	0.02	7
WM and general mathematics test	0.45	< 0.001	[0.34, 0.56]	1992.37 (12)	99.51	0.04	13

*CI* confidence interval, *gl* degrees of freedom, *k* total number of effect sizes, *n* number of studies, *FE* IC, CF, and WM combined, *IC* inhibitory control, *CF* cognitive flexibility, *WM* working memory

^a^all math tests together

*p < 0,001

**Fig. 2:**
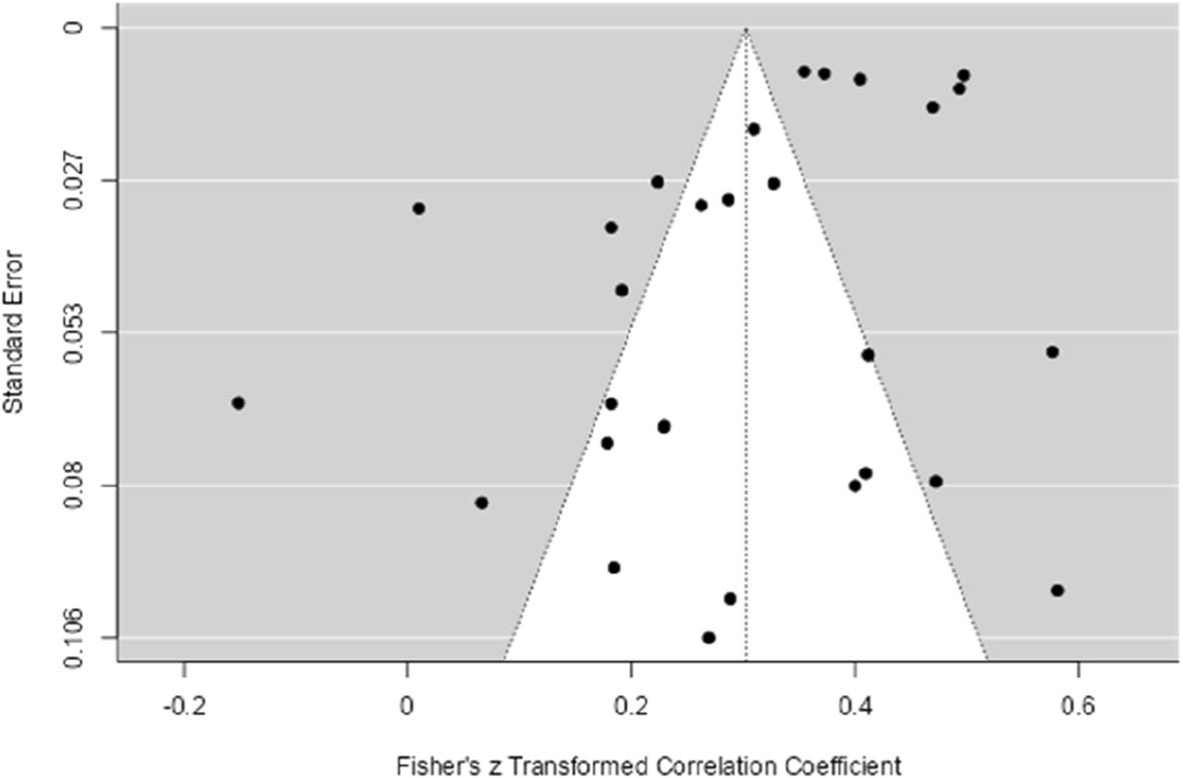
Funnel Plot with the Results of the General Analysis of the Relationship Between EF and Performance in Mathematics

**Fig. 3:**
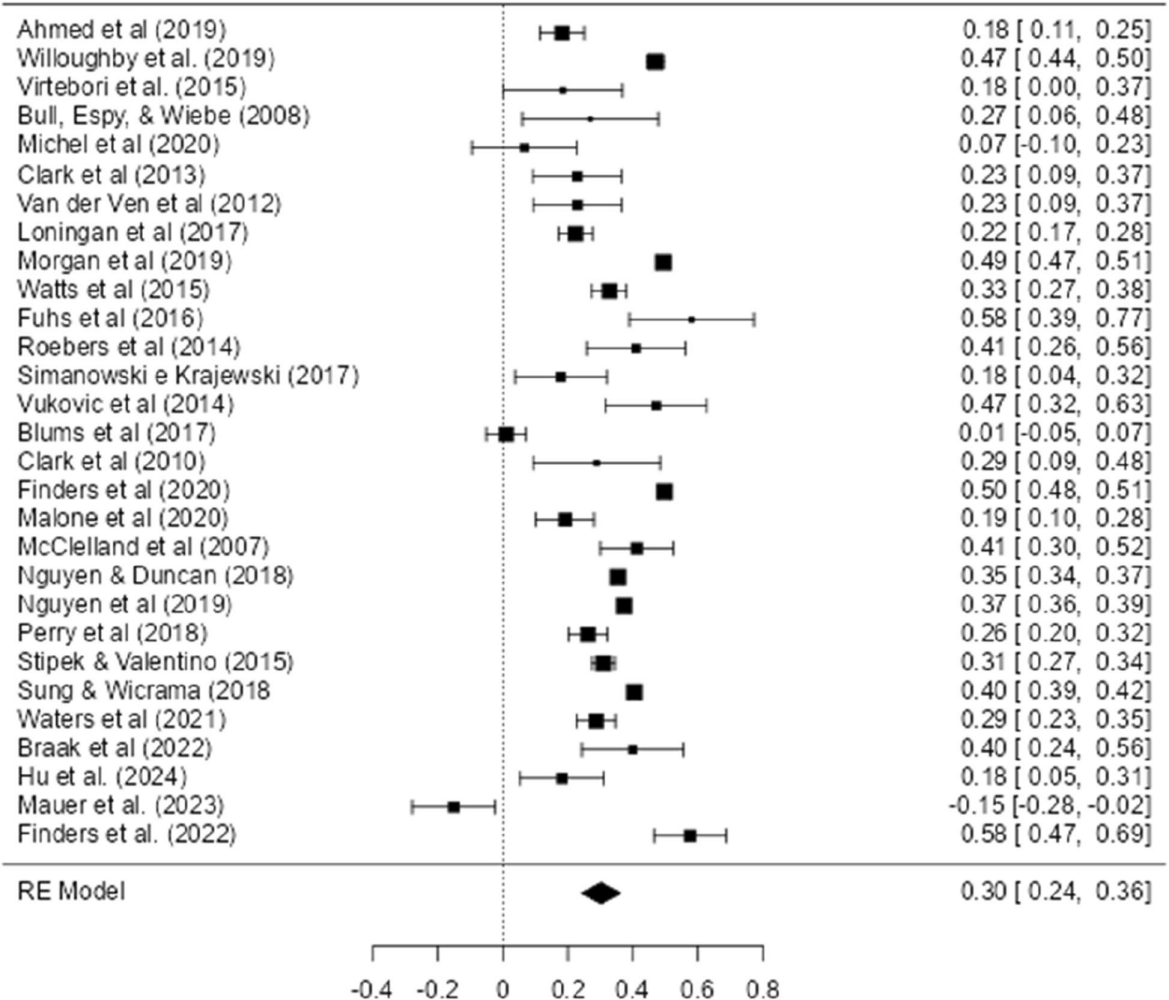
Forest Plot with EF Effect Sizes and Mathematics

Considering the possibility of publication bias, the funnel plot (see Fig. 2 and Fig. 3) showed a certain symmetry in the data, and Egger's regression test was not statistically significant (*p* = 0.30), suggesting a low influence of publication bias. Therefore, based on both analyses (funnel plot and Egger's test, see Table 3), the influence of publication bias in the studies analyzed is low.

**Table 3:** Assessment of publication bias between EF components and mathematics performance

Component	Egger's Test (p)	Funnel Plot
General Analysis: EF and Math^a^	0.30	symmetry
IC and Math^a^	0.50	symmetry
CF and Math^a^	0.34	symmetry
WM and Math^a^	0.002*	Asymmetry

*IC* inhibitory control, *CF* cognitive flexibility, *WM* working memory

^a^all math tests together

*p < 0,05

For the meta-regression analysis, which included'age'as a moderator, the studies by Sung and Wickrama (2018) were removed, as they did not include the children's ages. The meta-regression model was not significant for initial age (B = −0.001; 95% CI [−0.005, 0.003], *p* = 0.60), nor for final age (B = −0.001; 95% CI [−0.003, 0.001], *p* = 0.32). Thus, neither the initial nor final age were moderators of the relationship.

#### Analysis considering each EF component

##### IC and performance in mathematics

**General Analysis: IC and all Mathematics Tests**

**Fig. 4:**
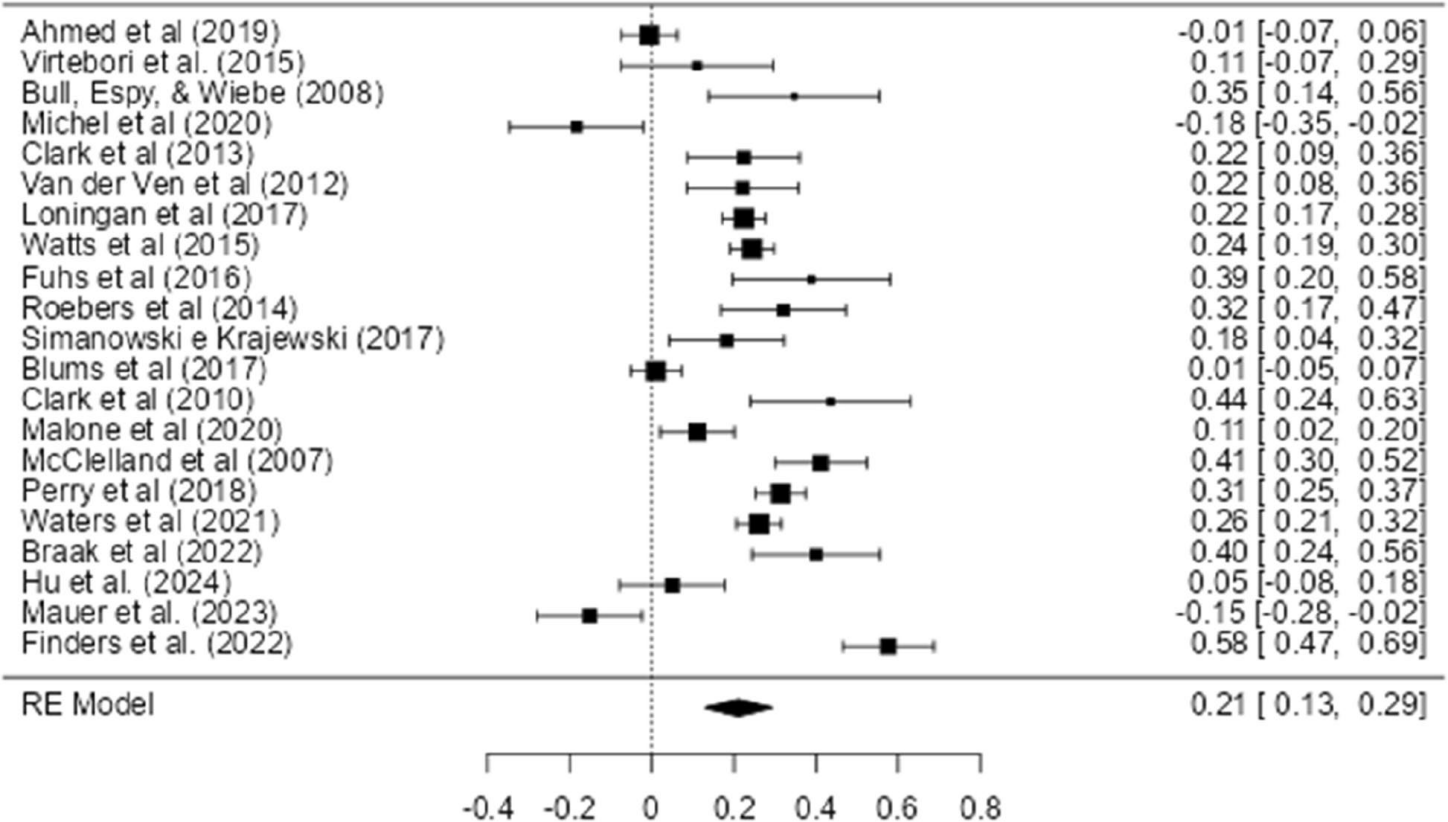
Forest Plot with IC Effect Sizes and Mathematics

Figure 4 shows the Forest Plot with the effect sizes of the relationship between IC and mathematical ability. In this figure, most of the effect sizes (and their confidence intervals) are to the right of the vertical line (which signs the absence of an effect: *r* = 0.00), indicating a positive correlation between IC and mathematics.

As can be seen in Table 2, a meta-analysis was conducted using 21 correlations between IC and all math tests combined (when a study presented more than one correlation, the mean correlation was calculated to ensure that only one correlation per study was included in the analysis). The outcome of the meta-analysis resulted in high heterogeneity (*Q* (20) = 231.2, *Tau*^2^ = 0.03, *I*^2^ = 93.65%, *p* < 0.001), and the effect size obtained was small and significant (*r* = 0.21, CI 95% [0.13, 0.29]).

**Fig. 5:**
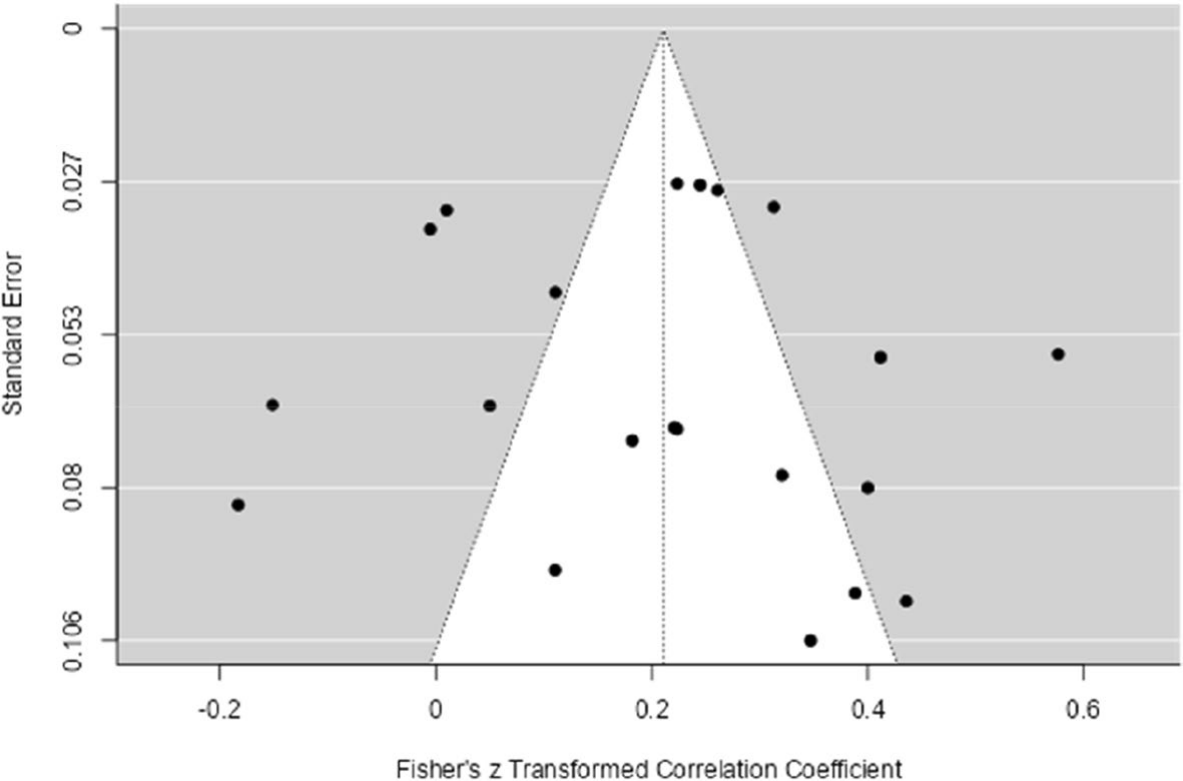
Funnel Plot with the Results of the General Analysis of the Relationship Between IC and Performance in Mathematics

The funnel plot (see Fig. 5) showed symmetrical data and Egger's regression test (*p* = 0.5) was not significant. Therefore, based on both analyses (funnel plot and Egger's test, see Table 3), the influence of publication bias in the studies analyzed is low.

The meta-regression model was not significant for the initial age (B = −0.003, CI 95% [−0.007, 0.002], *p* = 0.20), nor for the final age (B = −0.002, CI 95% [−0.005, 0.000], *p* = 0.5). Thus, neither the initial nor final age moderated the relationship between IC and math.

#### Analysis by type of math test: IC and calculation

As can be seen in Table 2, the subgroup analysis of 7 correlations (when a study presented more than one correlation, the mean correlation was calculated to ensure that only one correlation per study was included in the analysis) from seven studies to assess the relationship between IC and calculation indicated high heterogeneity (*Q* (6) = 42.5, *p* < 0.001, *Tau*^2^ = 0.03, *I*^2^ = 90.8%) and the effect size obtained was small and not significant (*r* = 0.09, 95% CI [−0.06, 0.24], *p* = 0.22). The meta-regression model was significant for initial age (B = −0.01, CI 95% [−0.02, −0.005], *p* = 0.001). Although the effect was significant, the magnitude of the effect was very small. In other words, for every one-year increase in age, the size of the effect decreases −0.01, suggesting little practical importance. For the final age, the effect was not significant (B = −0.008, CI 95% [−0.02, 0.002], *p* = 0.09).

#### Analysis by type of math test: IC and word-problem solving

As can be seen in Table 2, the subgroup analysis of 12 correlations (when a study presented more than one correlation, the mean correlation was calculated to ensure that only one correlation per study was included in the analysis) from 12 studies to assess the relationship between IC and word-problem solving indicated high heterogeneity (*Q* (11) = 227.04, *p* < 0.001, *Tau*^2^ = 0.03, *I*^2^ = 95.46%). The effect size obtained was small and significant (*r* = 0.24, CI 95% [0.13, 0.34], *p* < 0.001). The meta-regression model was not significant for the initial age (B = 0.001, CI 95% [−0.006, 0.008], *p* = 0.75), nor for the final age (B = −0.001, CI 95% [−0.004, 0.000], *p* = 0.11). Thus, neither initial nor final age moderated the relationship.

#### Analysis by type of math test: IC and general math tests

As can be seen in Table 2, the subgroup analysis of 8 correlations (when a study presented more than one correlation, the mean correlation was calculated to ensure that only one correlation per study was included in the analysis) from 8 studies to assess the relationship between IC and general math tests indicated low heterogeneity (*Q* (7) = 15.58, *p* = 0.29, *Tau*^2^ = 0.004, *I*^2^ = 64.54%%) and the effect size obtained was small and significant (*r* = 0.24, CI 95% [0.17, 0.30], *p* < 0.001). The meta-regression model was not significant for the initial age (B = −0.001, CI 95% [−0.004, 0.001, *p* = 0.14), nor for the final age (B = 

−0.0004, CI 95% [−0.003, 0.002], *p* = 0.75). Thus, neither initial nor final age moderated the relationship.

##### CF and Performance in Mathematics

**General Analysis: CF and All Mathematics Tests**

**Fig. 6:**
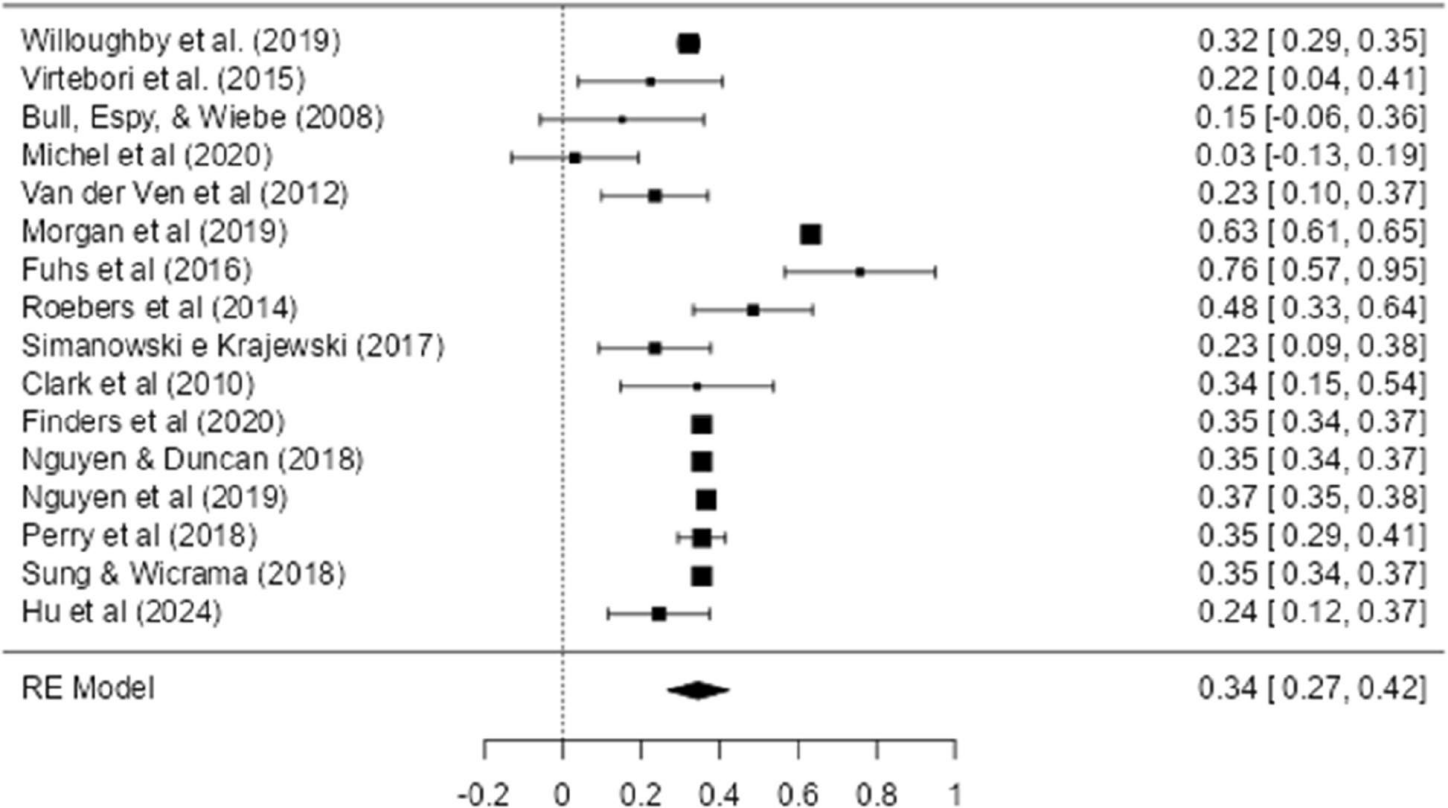
Forest Plot with CF Effect Sizes and Mathematics

Figure 6 shows the Forest Plot with the effect sizes of the relationship between CF and mathematical ability. This figure shows that most of the effect sizes (and their confidence intervals) are to the right of the vertical line (which signs the absence of an effect: r = 0.00), indicating a positive relationship between CF and mathematical ability.

As can be seen in Table 2, a general meta-analysis was conducted using 16 studies that included 16 correlations (when a study presented more than one correlation, the mean correlation was calculated to ensure that only one correlation per study was included in the analysis) between CF and all math tests. The result of the meta-analysis was highly heterogeneous (*Q* (15) = 674.96, *p* < 0.001, *Tau*^2^ = 0.02, *I*^2^ = 98.94%), and the effect size obtained was moderate and significant (*r* = 0.34, CI 95% [0.27, 0.42]).

**Fig. 7:**
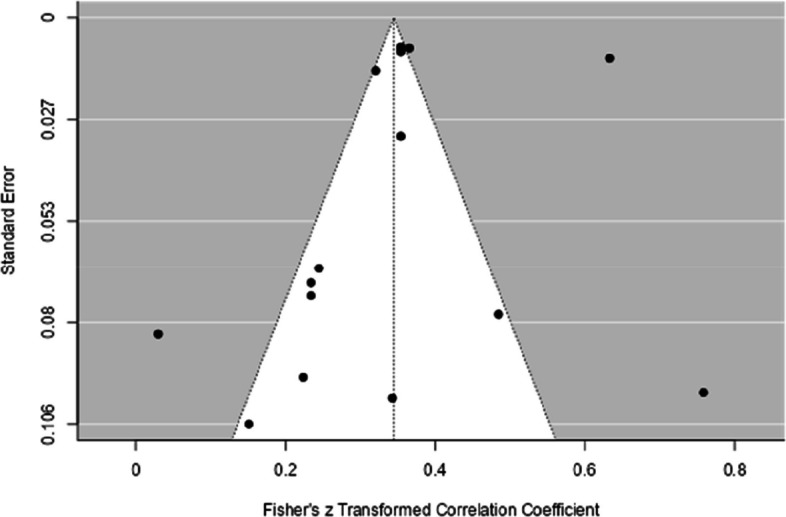
Funnel Plot with the Results of the General Analysis of the Relationship Between CF and Performance in Mathematics

As shown in Fig. 7, the funnel plot showed data symmetry, and Egger's regression test was not significant (*p* = 0.34). Therefore, based on both analyses (funnel plot and Egger's test, see Table 3), the influence of publication bias in the studies analyzed is low.

For the meta-regression analysis, which included age as a moderator, the studies by Sung and Wickrama (2018) were removed, as they did not include the children's ages in the study. The meta-regression model was not significant for the initial age (B = 

−0.0005, CI 95% [−0.006, 0.005], *p* = 0.84), nor for the final age (B = 0.0004, CI 95% [−0.005, 0.006], *p* = 0.94). Thus, neither the initial age nor the final age moderated the relationship.

#### Analysis by type of math test: CF and Calculation

As can be seen in Table 2, the subgroup analysis of 4 correlations (when a study presented more than one correlation, the mean correlation was calculated to ensure that only one correlation per study was included in the analysis) from 4 studies to assess the relationship between CF and calculation indicated low heterogeneity (*Q* (3) = 6.45, *p* = 0.09, *Tau*^2^ = 0.01, *I*^2^ = 53.66%). The effect size obtained was small and significant (*r* = 0.19, CI 95% [0.06, 0.31], *p* = 0.003). The meta-regression model was significant for the initial age (B = −0.012, CI 95% [−0.024, −0.001],* p* = 0.04). Although the effect was significant, the magnitude of the effect was very small. In other words, for every one-year increase in age, the size of the effect decreases 0.028, suggesting little practical importance. For the final age, the effect was not significant (B = −0.009, CI 95% [−0.023, 0.006], *p* = 0.24).

#### Analysis by type of math test: CF and word-problem solving

As can be seen in Table 2, the subgroup analysis of 4 correlations (when a study presented more than one correlation, the mean correlation was calculated to ensure that only one correlation per study was included in the analysis) from 4 studies to assess the relationship between CF and word-problem solving indicated high heterogeneity (*Q* (3) = 22.4, *p* = 0.001, *Tau*^2^ = 0.05, *I*^2^ = 92.11%) and the obtained effect size was moderate and significant (*r* = 0.40, CI 95% [0.16, 0.63], *p* = 0.001). The meta-regression model was not significant for the initial age (B = 0.027, CI 95% [−0.013, 0.068], *p* = 0.19), nor for the final age (B = 0.029, CI 95% [−0.074, 0.132], *p* = 0.39).

#### Analysis by type of math test: CF and general math tests

As can be seen in Table 2, the subgroup analysis of 10 correlations (when a study presented more than one correlation, the mean correlation was calculated to ensure that only one correlation per study was included in the analysis) from 10 studies to assess the relationship between CF and general math tests indicated high heterogeneity (*Q* (9) = 633.47, *p* < 0.001, *Tau*^2^ = 0.01, *I*^2^ = 98.99%) and the effect size obtained was moderate and significant (*r* = 0.36, CI 95% [0.28, 0.44], *p* < 0.001). The meta-regression model was not significant for the initial age (B = 0.003, CI 95% [−0.022, 0.030], *p* = 0.80), nor for the final age (B = 0.004, CI 95% [−0.005, 0.0013], *p* = 0.39).

##### WM and Performance in Mathematics

**General Analysis: WM and all Mathematics Tests**

**Fig. 8:**
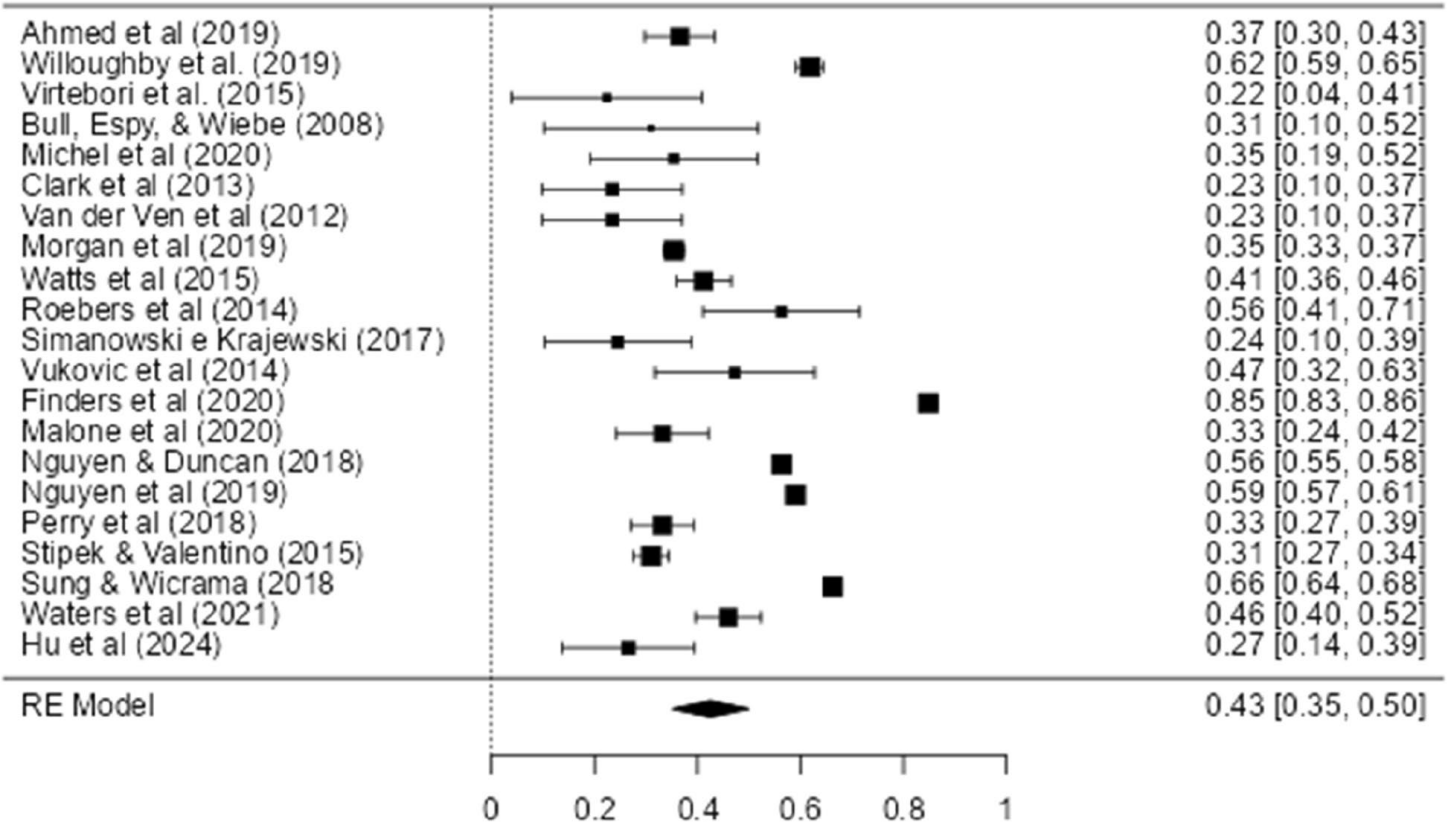
Forest Plot of the Effect Sizes of WM on Math Performance

Figure 8 shows the Forest Plot with the effect sizes of the relationship between WM and mathematical ability. In this figure, the effect sizes (and their confidence intervals) are to the right of the vertical line (which signs the absence of an effect*: r* = 0.00), indicating a positive relationship between WM and mathematics.

As can be seen in Table 2, a meta-analysis was conducted with 21 correlations (when a study presented more than one correlation, the mean correlation was calculated to ensure that only one correlation per study was included in the analysis) from 21 studies which assessed the relationship between WM and all math tests. The result of the meta-analysis was highly heterogeneous (*Q* (20) = 2140.6, *p* < 0.001, *Tau*^2^ = 0.03, *I*^2^ = 98.99%), and the effect size obtained was moderate and significant (*r* = 0.43, CI 95% [0.35, 0.50]).

**Fig. 9:**
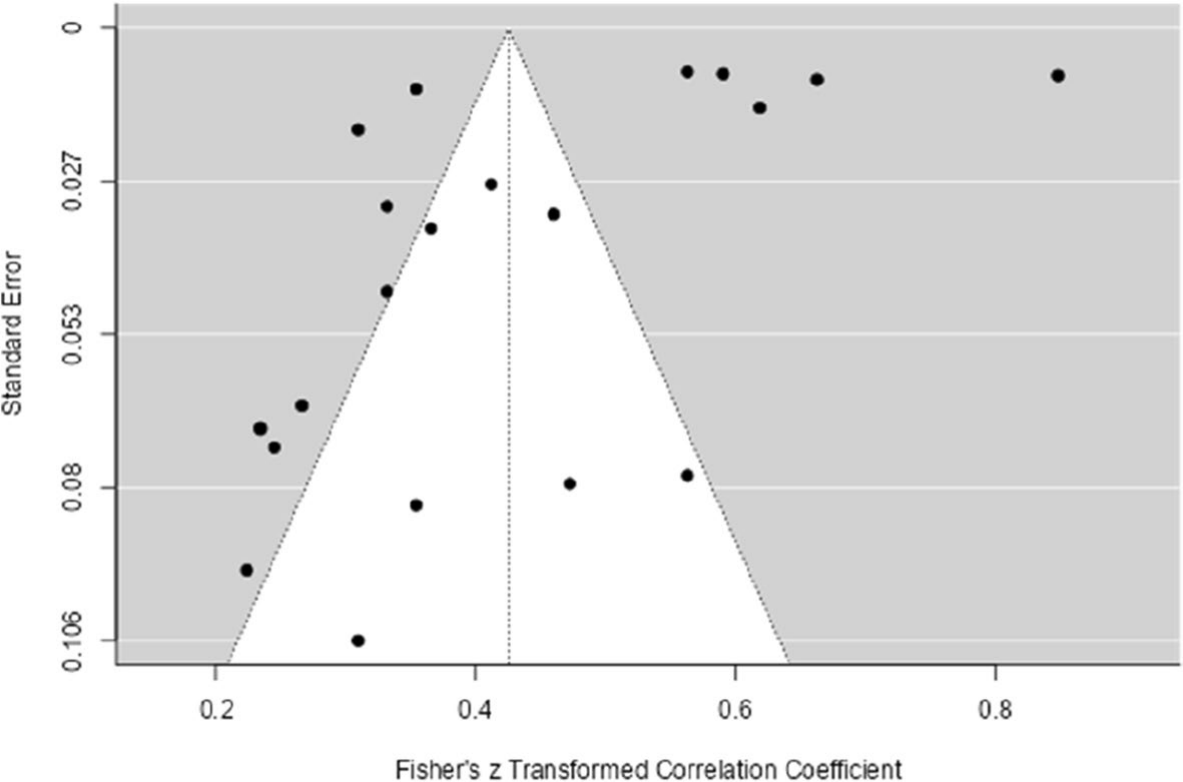
Funnel Plot with the Results of the General Analysis of the Relationship Between WM and Performance in Mathematics

The funnel plot, shown in Fig. 9, showed asymmetry in the data, confirmed by Egger's regression test (*p* = 0.002), indicating the possibility of publication bias. Therefore, it is not possible to rule out the hypothesis of publication bias in the studies analyzed.

For the meta-regression analysis, the studies by Sung and Wickrama (2018) were removed, as they did not include the children's ages in the study. The meta-regression model was not significant for the initial age (B = −0.0008, CI 95% [−0.005, 0.003], *p* < 0.70), nor for final age (B = −0.0003, CI 95% [−0.003, 0.002], *p* = 0.80). Thus, neither the initial age nor the final age moderated the relationship.

#### Analysis by type of math test: WM and calculation

As can be seen in Table 2, a meta-analysis was carried out containing 5 correlations (when a study presented more than one correlation, the mean correlation was calculated to ensure that only one correlation per study was included in the analysis) from 5 studies between WM and calculation. The results indicate low heterogeneity (*Q* (4) = 3.6, *p* = 0.47, *Tau*^2^ = 0.00, *I*^2^ = 0.05%), and the effect size obtained was moderate and significant (*r* = 0.35, CI 95% [0.29, 0.41]). The meta-regression model was not significant for the initial age (B = 0.007, CI 95% [−0.004, 0.0173], *p* = 0.23), nor for the final age (B = 0.002, CI 95% [−0.004, 0.009], *p* = 0.49).

#### Analysis by type of math test: WM and word-problem solving

As can be seen in Table 2, the subgroup analysis of 7 correlations (when a study presented more than one correlation, the mean correlation was calculated to ensure that only one correlation per study was included in the analysis) from 7 studies to assess the relationship between WM and word-problem solving indicated high heterogeneity (*Q* (6) = 50.59, *p* < 0.001, *Tau*^2^ = 0.02, *I*^2^ = 90.87%), and the effect size obtained was moderate and significant (*r* = 0.33, CI 95% [0.23, 0.43], *p* < 0.001). The meta-regression model was not significant for the initial age (B = 0.002, CI 95% [−0.003, 0.007], *p* = 0.46), nor for the final age (B = 0.001, CI 95% [−0.001, 0.003], *p* = 0.25).

#### Analysis by type of math Test: WM and general math tests

As can be seen in Table 2, the subgroup analysis of 13 correlations (when a study presented more than one correlation, the mean correlation was calculated to ensure that only one correlation per study was included in the analysis) from 13 studies to assess the relationship between WM and general math tests indicated high heterogeneity (*Q* (12) = 1992.37, *p* < 0.001, *Tau*^2^ = 0.04, *I*^2^ = 99.51%), and the effect size obtained was moderate and significant (*r* = 0.45, CI 95% [0.34, 0.56], *p* < 0.001). The meta-regression model was not significant for initial age (B = −0.0001, CI 95% [−0.007, 0.004], *p* = 0.56), nor for the final age (B = −0.0008, CI 95% [−0.007, 0.007], *p* = 0.78).

## Discussion

This study aimed to evaluate the relationship between EF and mathematics, considering longitudinal studies carried out with children or adolescents as primary data sources. The sample consisted of 29 studies, and the overall result of the meta-analyses indicates that the magnitude of the effect between EF and mathematics was moderate and significant (*r* = 0.30, CI 95% [0.24, 0.36]). It could be expected that the correlation found in this study would be weaker than that found in the other meta-analyses because this study, unlike the others, considered only asynchronous correlations and children and adolescents with typical development. However, the correlation found is very consistent with those reported by Spiegel et al., (2021, *r* = 0.36, CI 95% [0.33, 0.38]) and Pascual et al., (2019, *r* = 0.37, CI 95% [0.30, 0.42]). It is important to note that of the 29 studies included in this meta-analysis, 19 assessed EF at an interval equal to or greater than two years prior to the measurement of mathematics, thus indicating, in addition to the predictive power of EF, the stability of the relationship between EF and mathematics.

### The relationship between the different components of EF and mathematics

Considering the relationship between each of the EF components, they all showed significant correlations with the math measures, except the correlation between IC and calculation (r = 0.09, 95% CI [−0.06, 0.24] *p* < 0.22). Specifically, when all the math measures were analyzed together, WM showed the strongest correlation (*r* = 0.43, 95% CI [0.35, 0.50]). The fact that WM correlates more strongly with math performance than the other EF is consistent with the literature (Friso-van den Bos et al., 2013; Peng et al., 2016; Spiegel et al., 2021; Zhong et al., 2022). To be able to perform a math task, children need to store the arithmetic facts, retrieve the function of the mathematical operation, and thus cognitively perform the mathematical procedure to obtain the result, for example.

Although the correlation between CF and mathematics (*r* = 0.34, 95% CI [0.27, 0.42]) was weaker than the correlation between WM and mathematics, it is still a correlation of moderate magnitude, indicating that CF is also required to perform mathematical tasks. This result is also in line with other meta-analyses carried out with a sample of a similar age range to the present study (e.g., Yeniad et al., 2013, found *r* = 0.26, 95% CI [0.15, 0.35] and Santana et al., 2022, found *r* = 0.35, 95% CI [0.34, 0.36]).

Of the three EF components assessed in this study, IC showed the weakest correlation with mathematics (*r* = 0.21, 95% CI [0.13, 0.29]). This result is consistent with that found by Spiegel et al., (2021, *r* = 0.26; 95% CI [0.23, 0.29], age 5–12 years) and by Zhu et al., (2025, *r* = 0.19, 95% CI [0.14, 0.23, age 6–11 years]). The fact that Zhong et al. (2022) and Allan et al. (2014) found moderate correlations (*r* = 0.35 in both studies) may be due to the sample age of their studies since both were carried out with children up to 7 years of age and some studies indicate that the correlation between IC and mathematics is stronger among younger children than among older children (Bull et al., 2008; Simanowski & Krajewski, 2019; Viterbori et al., 2015), although the present meta-analysis found no evidence that age is a moderating variable in the relationship between IC and mathematics. Another possibility for the greater correlations observed in Zhong et al. and Allan et al. studies is that their studies were conducted with synchronous correlations; thus, the strength of the correlations tends to be greater because ic and math were measured at the same time. in any case, performance on ic tasks applied at time 1 correlated significantly with performance on math tasks applied later, indicating the importance of IC for mathematics as well.

### The Relationship between EF and different measures of performance in mathematics

#### WM and different measures of performance in mathematics

With regard to the possible moderating role of math tests in the relationship between WM and math performance, the present study found a statistical difference between the correlation of WM with calculation (*r* = 0.35, 95% CI [0.29, 0.41]) and with general math tests (*r* = 0.45, 95% CI [0.34, 0.56]), as well as between the correlation of WM with word-problem solving (*r* = 0.33, 95% CI [0.23, 0.43]) and with general math tests. However, there was no statistical difference between calculation and word-problem solving. Friso-van den Bos et al. (2013) point out that general math tests are often reported to correlate more strongly with WM than other specific math tests.

The finding of a correlation of the same magnitude between WM and calculation, and between WM and word-problem solving in the present study aligns with Peng et al. (2016). These authors found no significant difference between the correlation of WM and word-problem solving (*r* = 0.37, 95% CI [0.34 0.41]) and between WM and calculation (*r* = 0.35, 95% CI [0.32, 0.39]). However, this is inconsistent with the findings of Spiegel et al. (2021), as those authors found a slightly stronger correlation between WM and word-problem solving (*r* = 0.43, 95% CI [0.40, 0.47]) than between WM and calculation (*r* = 0.37, 95% CI [0.34, 0.40]). Despite these variations, all correlations are considered to be of moderate magnitude.

The type of math test influences the correlation between WM and math. Tests that require different mathematical skills, such as general math tests, demand more from WM since performance on these tests was correlated more strongly with WM than performance on calculation tests, consistent with the present results and those by Friso-van den Bos et al. (2013). One possibility is that general math tests tap on more diverse and complex math skills allowing for more variation in performance and requiring more from WM than calculation tests (in which, some answers can be retrieved by route, e.g., 2 × 3 = 6).

#### CF and different measures of performance in mathematics

As for the moderating role of math tests in the relationship between CF and math, the present study found no difference between the correlation of CF and calculation (*r* = 0.19, 95% CI [0.06, 0.31]) and the correlation between CF and word-problem solving (*r* = 0.40, 95% CI [0.16, 0.63]), as well as the latter and the correlation between CF and general math measures (*r* = 0.36, 95% CI [0.28, 0.44]). However, there was a statistical difference between the calculation and general math tests. Additionally, the magnitude of the correlation between CF and calculation is weaker compared to the correlation between CF and word-problem solving, as well as CF and general math tests.

Word-problem solving and general math tests will likely require the child to switch between mathematical and language skills. For example, in word-problem solving tests, the child will need to understand a verbally formulated problem and use procedural strategies to transform the problem into a mathematical calculation to be carried out. Of the meta-analyses previously carried out, the study by Spiegel et al. (2021) allows for better comparisons since the authors also considered the correlation between CF and measures of calculation and word-problem solving.

These authors did not find a significant difference in the correlations between CF and calculation (*r* = 0.29, 95% IC [0.19, 0.39]) and CF and word-problem solving (*r* = 0.35, 95% CI [0.22, 0.47]), and found a significantly weaker correlation of CF with measures of calculation fluency (*r* = 0.16, 95% CI [0.08, 0.23]). The results of the present study are consistent with those of Spiegel et al. (2021), except with regard to calculation fluency. It is important to highlight this result because, in the present meta-analysis, measures of calculation accuracy and fluency were combined, which may have contributed to the detection of a weaker correlation between CF and calculation compared to the correlation between CF and general math test.

#### IC and different measures of performance in mathematics

As for the moderating role of math tests in the relationship between IC and math, it can be said, based on the present study, that the types of math tests affect the relationship between IC and math since there was not a statistically significant correlation between IC and calculation (*r* = 0.09, 95% CI [−0.06, 0.24], p = 0.22, but there were statistically significant correlations between IC and word problem solving (*r* = 0.24, 95% CI [0.13, 0.34]), and between IC and general math tests (*r* = 0.24, 95% CI [0.17, 0.30]). This result is consistent with that reported by Friso-van den Bos et al. (2013), as these researchers found a stronger correlation of IC with general math measures.

### Age as a moderator of the relationship between EF and mathematics

This study found no moderating effect of age on the relationship between WM and mathematics, which is consistent with the study by Spiegel et al. (2021) and Zhu et. al. (2025). Peng et al. (2016) also found no moderating effect of age when analyzing the correlation between WM and a measure in which all math tests were considered together. However, the researchers found a stronger correlation between WM and calculation and geometry among younger children when considering specific math measures. An important point is that, in the study by Friso-van den Bos et al. (2013), a moderating effect of age was also not found when the researchers assessed the relationship between verbal WM and mathematics, unlike what was found when visuospatial WM was considered. Although the present study did not consider measures of verbal and visuospatial WM separately, most of the studies included in this meta-analysis used measures of verbal WM (see Appendix B), so the results of the present study are consistent with Friso-van den Bos et al. in this respect. In light of the literature and in line with this study, a body of evidence indicates that age is not a moderating factor in the relationship between verbal WM and mathematics.

This study also found no moderating effect of age on the relationship between CF and mathematics. The literature on this topic is conflicting, with Santana et al. (2022), for example, reporting a moderating effect of age and Yeniad et al. (2013) not. Generally, the magnitude of the effect is greater in younger children (Friso-van den Bos et al., 2013; Santana et al., 2022; Spiegel et al., 2021; Yeniad et al., 2013), even if the difference is not significant.

Regarding the association between IC and mathematics, as in several other meta-analyses (Allan et al., 2014; Friso-van den Bos, et al., 2013; Spiegel et al., 2021), no moderating effect of age was observed in the present study. At first, it was thought that age would moderate the relationship between EF and mathematics, especially since only longitudinal studies covering ages from birth to 18 were included. However, this was not observed. The first hypothesis for this result is the possibility that there is, in fact, a constant contribution of EF to mathematics throughout development. As Spiegel et al. (2021) mentioned, mathematical tasks have an inherent complexity that remains constant throughout development. Once one mathematical skill is developed (addition), another is introduced (subtraction), and the demand for EF remains constant. A second hypothesis is related to the characteristics of the studies included. Considering that few studies have included samples with adolescents (Ahmed et al., 2019; Stipek & Valentino, 2015; Watts et al., 2015), it is assumed that not enough variability was observed to obtain a significant effect of age. In general, the moderating role of age in the relationship between EF and mathematics should not be completely ruled out; however, this study, in conjunction with the previous literature (Friso-van den Bos et al., 2013); Spiegel et al., 2021; Peng et al., 2016; Yeniad et al., 2013; Zhu et al., 2025) seems to indicate that the association of EF with mathematical skills is not moderated by age.

### Limitations and suggestions for future studies

This study has some limitations. Firstly, high heterogeneity was observed in most analyses, which may indicate that other covariates moderate the relationship between EF and mathematics. This high heterogeneity is also observed in other meta-analyses (Friso-van den Bos et al., 2013; Pascual et al., 2019; Spiegel et al., 2021; Yeniad et al., 2013; Zhong et al., 2022). Some moderating variables were not assessed in this study, such as gender, which has been significant in other studies (Pascual et al., 2019; Zhong et al., 2022). However, the included studies did not provide individual correlations for this variable. In addition to this, another variable that potentially moderates the relationship between EF and mathematics, which was not included in the present study, refers to the instruments used to assess EF. Zhong et al. (2022) found that this variable moderated the relationship between EF and mathematics. According to these researchers, although the different versions of the Stroop Task, for example, are very similar in general terms, they differ in content, which can affect the results. Another important limitation of this study is the exclusion of studies that did not report correlations between executive functions at Time 1 (T1) and mathematics at Time 2 (T2). This decision was partly guided by the need for consistency with previous meta-analyses that followed the same approach. However, this strategy may have led to the exclusion of relevant studies, resulting in a potential loss of data. Nevertheless, It is important to notice that, in the present study, of the 20 studies excluded for not reporting longitudinal correlations, 18 would have been excluded for other methodological reasons as well—for example, not reporting effect size measures, combining data from typically and atypically developing children, aggregating data across all time points, or administering math tests only at Time 1. Thus, the application of this criterion resulted in the exclusion of only two studies that presented analyses other than correlations. Nevertheless, limiting the present meta-analysis to correlations restricts the conclusions about the longitudinal relationships between executive functions and mathematics. Another possible limitation is the search terms used in the present study. We opted for broad terms, such as executive function and math, to be as inclusive as possible. Although it is unlikely that a study using a more specific term, such as"shifting"and"self-regulation,"would not use"executive function"in the title, abstract, or keywords, it is a possibility; therefore, such a study would not be retrieved.

As a suggestion for future studies, in addition to including the analysis of more moderating variables (e.g. gender, types of EF tests, accuracy vs. speed in mathematical tasks, verbal WM vs. visuospatial WM) in meta-analysis studies investigating the relationship between EF and mathematics, it is also vital that primary data reports provide precise information on gender, mean age of the sample with standard deviation, sample size, nationality of the sample, distribution of the variables and reliability of the measures in the method section, as this helps researchers wishing to carry out review and meta-analysis studies.

Given that no study has been found in Latin America that has longitudinally investigated the relationship between EF and mathematics, future studies could fill this gap as the particularities of each region have the potential to impact cognitive variables.

## Conclusion

This study is the first meta-analysis to investigate the relationship between the three components of executive function (EF) and mathematics, based solely on longitudinal studies. The results of this study indicated that EF and its subcomponents (i.e., IC, WM, and CF), measured at Time 1, significantly predicted the mathematical abilities subsequently assessed. Although publication bias cannot be completely ruled out in this study, two statistical techniques were implemented to analyze it, and it does not appear to be a validity problem in the current study. Thus, based on these results, screening for deficits in any of the three components of EF is recommended, aiming to provide children with opportunities for support or intervention as early as possible in order to benefit from the greater neuroplasticity present at earlier stages of development and avoid the cascading effect of deficits in EF, such as difficulties with math.

## Supplementary Information

Supplementary Material 1

Supplementary Material 2

## Data Availability

The datasets generated are available in the Open Science Framework repository: https://osf.io/hck4s/?view_only=d4bdcc5a5f60463198741ea3b749cdb2

## Abbreviations

CF: Cognitive Flexibility
CI: Confidence Interval
EF: Executive Function
IC: Inhibitory Control
M: Mean
SD: Standard Deviation
WM: Working Memory

## Footnotes

1 This sample size was estimated using the G*Power3 software, considering 95% of power to detect a bivariate correlation of 0,3 with a one-tailed *p* = 0,05.
